# Evidence from glycine transfer RNA of a frozen accident at the dawn of the genetic code

**DOI:** 10.1186/1745-6150-3-53

**Published:** 2008-12-17

**Authors:** Harold S Bernhardt, Warren P Tate

**Affiliations:** 1 Department of Biochemistry, Otago School of Medical Sciences, University of Otago, Dunedin, New Zealand

## Abstract

**Background:**

Transfer RNA (tRNA) is the means by which the cell translates DNA sequence into protein according to the rules of the genetic code. A credible proposition is that tRNA was formed from the duplication of an RNA hairpin half the length of the contemporary tRNA molecule, with the point at which the hairpins were joined marked by the canonical intron insertion position found today within tRNA genes. If these hairpins possessed a 3'-CCA terminus with different combinations of stem nucleotides (the ancestral operational RNA code), specific aminoacylation and perhaps participation in some form of noncoded protein synthesis might have occurred. However, the identity of the first tRNA and the initial steps in the origin of the genetic code remain elusive.

**Results:**

Here we show evidence that glycine tRNA was the first tRNA, as revealed by a vestigial imprint in the anticodon loop sequences of contemporary descendents. This provides a plausible mechanism for the missing first step in the origin of the genetic code. In 448 of 466 glycine tRNA gene sequences from bacteria, archaea and eukaryote cytoplasm analyzed, CCA occurs immediately upstream of the canonical intron insertion position, suggesting the first anticodon (NCC for glycine) has been captured from the 3'-terminal CCA of one of the interacting hairpins as a result of an ancestral ligation.

**Conclusion:**

That this imprint (including the second and third nucleotides of the glycine tRNA anticodon) has been retained through billions of years of evolution suggests Crick's 'frozen accident' hypothesis has validity for at least this very first step at the dawn of the genetic code.

**Reviewers:**

This article was reviewed by Dr Eugene V. Koonin, Dr Rob Knight and Dr David H Ardell.

## Background

Di Giulio has argued persuasively that tRNA evolved from the duplication of a hairpin half the length of the contemporary molecule, based on the homology between the 5' and 3' halves of tRNA [1,2]. This has been supported by a statistical analysis of the 5' and 3' halves of contemporary tRNA by Widmann *et al*. (2005) which concluded that their results 'support the hypothesis that the modern tRNA cloverleaf arose from a single hairpin duplication prior to the divergence of modern tRNA specificities and the three domains of life' [3]. Di Giulio has suggested that this hairpin origin has been preserved in the division of some contemporary tRNA genes into two 'halves' by the canonical intron insertion point between positions 37 and 38 in the anticodon loop, just one nucleotide downstream of the anticodon triplet [1].

**Figure 1:**
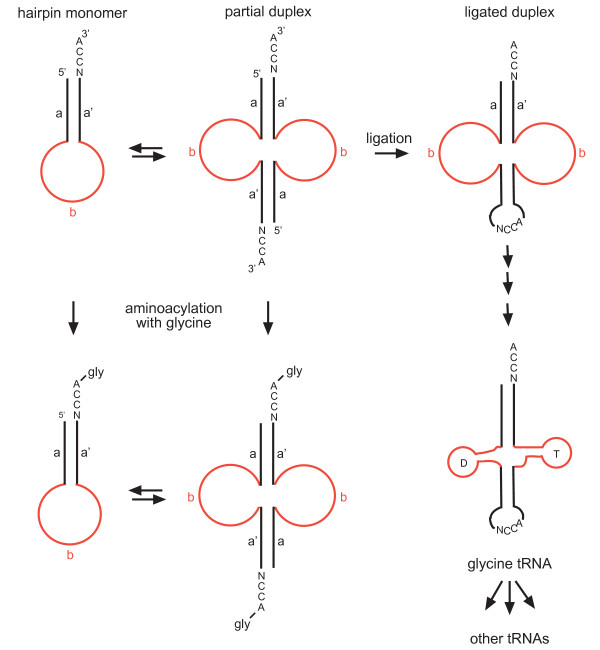
**Proposed hairpin duplication origin of tRNA**. Hairpin monomer (*top left*) is in equilibrium with partial duplex (*top middle*), both of which are able to be specifically aminoacylated with glycine by the RNA predecessor of contemporary glycyl-tRNA synthetase (*bottom left and middle*). The defining moment in the origin of tRNA was the ligation of the partial duplex, which created a covalently joined molecule (*top right*), anticodon loop, and anticodon from the 3'-terminal CCA sequence of the upstream hairpin [1,2,32]. Mutations (principally in the central loops) produced the precursor to contemporary glycine tRNA (*bottom right*). Subsequent duplication and mutation to re-evolve the amino acid-specific RNA operational code sequences of the other amino acid-accepting hairpins (with accompanying mutation of the anticodon) led to a proliferation of tRNA sequences and, eventually, coded protein synthesis.

From the experiments of Schimmel [4] it appears possible that, prior to the emergence of coded protein synthesis, there existed up to eleven hairpins with 3'-CCA termini and particular stem nucleotides, allowing for specific aminoacylation (as has been demonstrated for hairpin analogues of eleven contemporary tRNAs; see Table 1, *middle column*). Aminoacylated hairpins might have participated in noncoded protein synthesis [5], or the hairpins could have acted as 'handles' for amino acids that functioned as cofactors [6], or, possibly, both could have been true. Each of the hairpins was likely in equilibrium with its duplex due to the symmetry of hydrogen bonding interactions. As shown in Figure 1, these partial duplexes appear to be the forerunners of tRNA. Or, as Di Giulio has noted, '...hairpin structures are apparently such close precursors to tRNA molecules that, in a sense, they imply them' [7]. (Interestingly, in contemporary biology hairpin structures of RNA give rise to many functional entities, such as protein binding motifs in mRNA and precursors to microRNAs.) However, the decisive event in the formation of the first tRNA was almost certainly the *ligation *of the two hairpins (Figure 1, *top right*).

**Table 1:** Proposed order of amino acid incorporation into genetic code, hairpin aminoacylation and stereochemical relationship with anticodon

	Amino acids in proposed order of incorporation into genetic code	Reported hairpin amino-acylation	Established stereochemical relationship with anticodon
	glycine	+	
	alanine	+	
	aspartic acid	+	
**early**	valine	+	
	proline		
	serine	+	
	glutamic acid		
	threonine		
	leucine		+
	arginine		codon
	asparagine		
	isoleucine	+	anticodon/codon
	glutamine	+	-
	histidine	+	+
	lysine		
	cysteine	+	
**late**	phenylalanine		+
	tyrosine	+	+
	methionine	+	
	tryptophan		+

*Left column*: proposed order of incorporation of amino acids into genetic code taken from [9]. *Middle column*: hairpin aminoacylation data taken from [4]. *Right column*: stereochemical relationship with anticodon data taken from [27]. Gaps in the table represent those examples where evidence has not been reported or established.

It is widely believed that the contemporary standard genetic code has evolved from a simpler predecessor, and that only a subset of the current 22 amino acids were originally encoded [8]. On the basis of an analysis of theories of the origin of the genetic code, Trifonov [9] has produced a 'league table' of when amino acids were incorporated into the code, extending from the likely earliest to the latest (providing a clue to which was the earliest amino acid, Trifonov had previously found glycine to be the most frequent amino acid amongst matching residues in probable ancestral sequences of homologous proteins between prokaryotes and eukaryotes [10]). The amino acids listed according to Trifonov's proposed incorporation into the genetic code from earliest to latest are shown in Table 1, *left column*. This order has been given some support by a statistical analysis by Jordan *et al*. [11] of the relative rate of amino acid gain/loss in orthologous proteins, where six out of eight amino acids found to be decreasing in frequency are those predicted by Trifonov [9] to have been incorporated into the genetic code at an earlier stage, and eight out of twelve found to be increasing in frequency [11] are those predicted to have been recruited later [9].

In previous work, we have found suggestive evidence of a residual CCA sequence in the anticodon loop sequences of contemporary glycine tRNAs, indicative of a hairpin duplication origin [12]. In order to confirm this preliminary finding we have analyzed the glycine tRNA gene sequences from two online databases.

## Results and discussion

We have analyzed 466 glycine tRNA gene sequences from bacteria, archaea and eukaryote cytoplasm taken from Sprinzl and Vassilenko [13] (see Methods section for further details). Of these, 96% (448) have the sequence CCA occurring immediately upstream of the canonical intron insertion position. We have also analyzed > 200 mitochondrial and chloroplast glycine tRNA gene sequences taken from [13] and [14]. Of these, almost 100% (208 of 209) have the sequence CCA occurring immediately upstream of the canonical intron insertion position. The data are consistent with our starting hypothesis [12] that the first anticodon (NCC for glycine) was derived from the 3'-terminal CCA of one of the interacting hairpins as a result of an ancestral ligation event (Figure 1), and imply that an 'accident' [8] lies at the heart of the genetic code (see below).

As shown in Figure 1 (*top left and middle*), in our proposal two hairpins would be in monomer-dimer equilibrium before the seminal ligation event fixed them in the duplex form. Each form could be aminoacylated with glycine by an RNA predecessor of a modern aminoacyl tRNA synthetase (Figure 1, *bottom left and middle*). The ligated duplex glycine RNA evolved into the modern glycine tRNA, but in this proposal has also experienced expanded evolution by duplication and mutation to give rise to the other tRNAs (*right bottom*).

**Figure 2:**
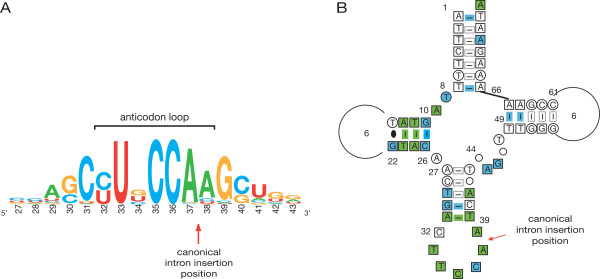
**Glycine tRNA consensus sequences showing canonical intron insertion position**. **(A) **Anticodon arm consensus sequence from 466 glycine tRNA genes from eubacteria, archaea and eukaryote cytoplasm taken from [13]. Note: T has been changed to U for the purpose of showing RNA sequence. Adapted from an image generated by WebLogo software [40]. **(B) **Cloverleaf consensus sequence of 136 mammalian mitochondrial glycine tRNA gene sequences taken from [14]. In the anticodon loop: conserved in 100% of sequences designated by green squares (*e.g*. C_35_, A_37_); in > 90% of sequences by blue squares (*e.g*. C_36_); in > 50% of sequences by the open square. Note: 3'-CCA terminus is not displayed. T has not been changed to U in this depiction.

A consensus sequence of the anticodon arm derived from the 466 bacterial, archaeal and eukaryote cytoplasm glycine tRNA gene sequences taken from [13], together with a cloverleaf representation of the consensus sequence of 136 mammalian mitochondrial glycine tRNA genes taken from [14] are shown in Figure 2. Figure 2A gives the nucleotide frequency at positions 27–43 of the modern glycine tRNA gene sequences from bacteria, archaea and eukaryote cytoplasm. The conservation of the anticodon loop at positions 35 and 36 of the anticodon itself, and position 37 (adjacent to the canonical intron insertion point) are clearly indicated. The conservation of U33 is almost certainly due to its role in forming the characteristic U-turn structure of the anticodon loop [15]. Figure 2B shows the conservation in the anticodon loop of the more restricted subset of the mammalian mitochondrial glycine tRNAs [14] (100% conserved in positions 33–38 encompassing the anticodon and intron insertion position, except for position 36 where perfect conservation is disrupted by a single sequence indicated to be from *Physeter catadon *(sperm whale), which has a UCU anticodon; this expands glycine codons to include AGA and AGG, in the standard code the codons for arginine). While more significant variation within the isolated subgroup of mitochondrial and chloroplast tRNAs would not have negated our proposal, it is compelling that this has not occurred.

**Figure 3:**
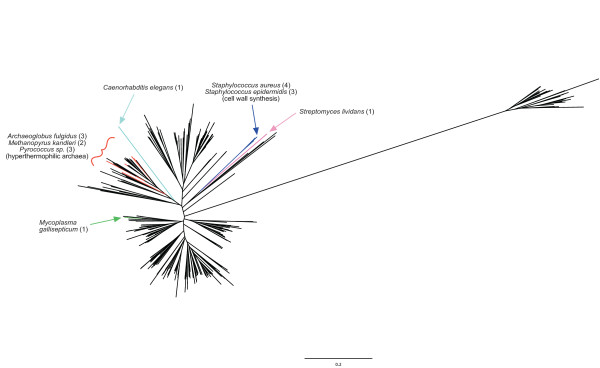
**Phylogenetic tree of glycine tRNA gene sequences from bacteria, archaea and eukaryote cytoplasm**. The phylogenetic relaxed neighbour joining tree was constructed by using 466 glycine tRNA gene sequences from eubacteria, archaea and eukaryote cytoplasm, taken from [13]. Branches including tRNA gene sequences *not *containing an anticodon loop CCA sequence are shown in colour, and indicated by the particular labels (with number of sequences in brackets).

In order to investigate further the antiquity of the CCA sequence in the anticodon loop of glycine tRNA, a phylogenetic analysis was carried out using the 466 glycine tRNA gene sequences from bacteria, archaea and eukaryote cytoplasm taken from [13]. Figure 3 shows a representative phylogenetic relaxed neighbour joining tree. It was not possible to identify an ancestral glycine tRNA gene sequence, but despite this the results were intriguing and suggestive. The tRNA gene sequences *not *containing an anticodon loop CCA are localized on isolated branches rather than spread throughout the tree, suggesting the loss of the CCA sequence is more likely a derived rather than ancestral character. The loss of this sequence in two sets of tRNAs can be explained by independent events. Firstly, in 7 sequences from *Staphylococcus aureus *and *Staphylococcus epidermidis *(Figure 3, *blue branch*), a subset of glycine tRNAs from these two species, A37 has been replaced by a pyrimidine. If this substitution has the effect of weakening the anticodon-codon interaction, it may have functioned to exclude these tRNAs from being used in ribosomal protein synthesis consistent with their involvement now in non-ribosomal protein synthesis of the bacterial cell wall [16] (it has been proposed that a purine in position 37 functions to stabilize the adjacent anticodon-codon interaction through base stacking on to the anticodon-codon helix [17]). Secondly, in 8 sequences from the hyperthermophilic archaeal species *Archaeoglobus fulgidus*, *Methanopyrus kandleri *and *Pyrococcus sp*. (Figure 3, *red branches*), A37 has been replaced by G, perhaps because of a general increase in G+C content in the tRNA and rRNA of these species [18]. There are three other sequences not containing an anticodon loop CCA that appear to be isolated examples, but in these cases their origin is unclear.

Schimmel's experimental work [4] suggests up to eleven aminoacylated hairpins (including one specific for glycine) might have been in existence prior to the advent of the tRNA molecule (Table 1, *middle column*). Why should glycine have been the first amino acid encoded? The assignment may have been made due to the relative prevalence of the various amino acids on the prebiotic, and possibly even *biotic *earth. Although the nature of the primordial earth's atmosphere is controversial, in recent experiments Miller and colleagues have shown that electric discharge stimulation even within an atmosphere containing 5% O_2 _(in 47.5% N_2_/47.5% CO_2_) produces small quantities of amino acids 'consisting almost entirely of glycine and racemic alanine', if analyzed in the presence of an antioxidant [19]. Why should the codons GGN (complementary to the NCC anticodons) have been the first assigned codons? As suggested by Eigen and Schuster, the GGN codons/NCC anticodons may have been selected first due to the relative strength of the G-C hydrogen bonding interaction [20].

What might have caused the ligation event? As shown in Figure 1 (*top middle*), the ends of the partial duplex would have been juxtaposed. Therefore, ligation may have occurred spontaneously, possibly catalyzed by a coordinated Mg^2+ ^ion. However, we suspect that the ligation was catalyzed by an ancestor of a self-splicing group I or II intron, for the following reason. The conservation of an intron insertion site between different organisms is often taken to imply descent from a common ancestor. For example, self-splicing group I introns found at the same position in leucine tRNAs in chloroplasts and cyanobacteria are taken as supporting the bacterial origin of these plastids [21,22]. Therefore, it seems reasonable to suppose that protein-spliced tRNA introns, usually found between nucleotides 37 and 38 in contemporary eukaryotes and archaea, have likewise descended from a common ancestor, possibly a group I or II intron that was located at this same position and was involved in the ancestral ligation event. This is consistent with the proposal that self-splicing group I and (possibly) group II tRNA introns are the most ancient introns, perhaps arising some 3,500 million years ago [23].

Regardless of the mechanism, ligation of the two hairpins would have been a critical step for the creation of a proto-tRNA molecule, proto-anticodon loop and (possibly at a later point) the anticodon itself. As a result of this seminal event, a *single *proto-tRNA was formed, which subsequently went on to re-evolve the sequences of the other hairpins already present, possibly by a process of duplication and mutation (Figure 1, *bottom right*). The idea of a 'bottleneck' through which only a single proto-tRNA passed has also been made by Wolf and Koonin, and explains the observation that contemporary tRNAs appear to be descended from a single ancestral molecule [24]. Others have also concluded that tRNA has evolved from a single ancestral molecule, including Ellington *et al*. [25] and, as previously discussed, Widmann *et al*. [3], although this is not a view shared by Di Giulio [26].

Our observations and proposal clearly have major implications for the origin of the genetic code, of which there are five main theories:

1. The stereochemical hypothesis holds that the assignment of anticodons/codons to amino acids was based on stereochemical affinity, that is, specific anticodon/codon sequences bind to specific amino acids [8]. Although the route from such an association to the structure of tRNA, with its widely separated anticodon and amino acid binding site, is far from clear [25], the theory has gained ground with the discovery of anticodon and/or codon sequences in putative amino acid binding sites of *in vitro *selected RNA aptamers. A correlation has been found for seven of eight amino acids tested, with aptamers binding glutamine not exhibiting this correlation [27] (Table 1, *right column*). It is perhaps significant that so far no aptamers have been found that bind the 'earliest' amino acids (a point also made by Wolf and Koonin [24]), although it has been pointed out that achieving specific binding of aptamers to a small amino acid such as glycine could be difficult, due to the simplicity of its R group (H) and lack of unique chemical groups with which it might present as a binding motif. Naturally occurring glycine-binding riboswitches have been discovered however in a variety of bacterial species in the 5'-UTR of the *gcvT *operon that contains genes involved in glycine degradation [28]. The conserved sequences of these riboswitches contain no glycine anticodon sequences. While a single glycine *codon *is found at one of the binding sites, two of its three nucleotides are part of a stem structure and so not immediately available for direct interaction with the amino acid [28].

2. The coevolutionary hypothesis holds that codons have been reassigned to 'new' amino acids on the basis of biosynthetic descent from precursor amino acids, with Wong suggesting by this reasoning that glycine, alanine, aspartic acid, glutamic acid and serine were the earliest amino acids [29].

3. The adaptive hypothesis proposes that the genetic code has evolved in such a way so as to minimize the effects of mutation, with the result that similar amino acids have related codons [30].

Knight *et al*. [31] have argued that these three theories (above) are not necessarily mutually exclusive, and each could have resulted in a code where chemically similar amino acids are assigned to related codons. Certainly, the coevolutionary and adaptive hypotheses concern the code's evolution rather than its origin, and thus are compatible with our theory of a 'frozen accident' at the dawn of the genetic code.

4. Others [*e.g*. [4,32-35]] have postulated an ancestral link between the genetic code and the operational RNA code, which occurs in the acceptor stem of contemporary tRNAs [4]. This code, which has been termed the second genetic code [36], comprises the three to four terminal base pairs of the stem plus the adjacent discriminator base, and determines specific aminoacylation by contemporary aminoacyl-tRNA synthetases. This code is the basis of Schimmel's work with aminoacylatable hairpins [4]. It is possible that as part of the hairpin duplication event that produced tRNA, the ancestral operational RNA coding sequence was duplicated and subsequently evolved to become both the contemporary operational RNA code sequence and the anticodon. Rodin *et al*. [33-35] identify the first three positions of the acceptor stem of contemporary tRNA as being homologous to the anticodon triplet even though, as they themselves admit, 'straightforward analysis failed to uncover any traces of homology in this case' [35]. Despite this, they have produced a mechanism for the elaboration of the genetic code by the production of tRNAs containing complementary anticodons. Again, while we believe such a mechanism could have been involved in the code's *evolution*, the question of how the code *began *still remains unanswered. We support the premise that the hairpin duplication origin of tRNA holds the key to the origin of the genetic code, but from a slightly different perspective. As mentioned, on the basis of homologies with contemporary glycine tRNAs, we believe the first anticodons were derived from the 3'-terminal CCA of the upstream hairpin rather than from the adjacent ancestral operational RNA code sequence.

5. Crick's 'frozen accident' hypothesis was originally presented as an explanation for the 'universality' of the genetic code, although at the time he proposed it he acknowledged the possibility of some natural variation [8]. This variation and its extent, particularly in mitochondria, has become evident only more recently [37]. In its extreme form the frozen accident theory proposes that the assignment of codons/anticodons to amino acids was completely random, but once made was locked in place due to the hugely deleterious effect any changes would have had on cell proteins. However, this extreme form of the theory is not supported by a non-standard code being found in organisms like the yeast *Candida albicans *[38] and the ciliates *Tetrahymena thermophila *and *Paramecium tetraurelia *[39]. Although it is now widely accepted that the genetic code as a whole is not frozen and at least partially free to evolve, perhaps this is not true for all sections of the code. Our work suggests the NCC glycine anticodons (derived from the 3'-CCA hairpin terminus) have been retained through billions of years of evolution. In addition, A37 (also part of the anticodon loop CCA sequence; see Figure 2A) has been retained, due perhaps, as previously discussed, to its ability to stabilize the adjacent anticodon-codon interaction (this position is normally occupied by either A or G [15]).

Finally, the conclusion reached from our study that glycine was the first amino acid incorporated into the genetic code is in agreement with Trifonov's analysis of ancestral protein sequences [10] and comprehensive review of the various and varied theories of the origin of the genetic code [9], Miller's experimental findings [19], and Jordan *et al.'s *analysis of amino acid gain/loss in contemporary proteins [11].

## Conclusion

The origin of the genetic code has attracted many theorists and resulted in almost as many theories for healthy and vigorous debate. Our hypothesis speaks to the seminal first step and is dependent upon tRNA originating from the ligation of RNA hairpins with 3'-CCA termini. The ligation was likely carried out by an ancestor of a self-splicing group I or II intron, with the ligation point having been retained as the most common position for protein-spliced introns in contemporary archaeal and eukaryote tRNAs. In terms of this framework, we have uncovered a previously overlooked feature of contemporary tRNA sequences that potentially explains the very first step in the origin of the genetic code. That this feature has been retained over four billion years of evolution supports Crick's hypothesis of a frozen accident, at least for this first step in the origin of the genetic code. Our theory has an elegant simplicity for providing the code's first 'stake in the ground' and provides a clear link between the anticodon loop CCA sequence found in nearly all contemporary glycine tRNA molecules and the proposed mechanism of hairpin duplication.

## Methods

### tRNA databases

The analysis of glycine tRNA gene sequences from bacteria, archaea and eukaryote cytoplasm used the Compilation of tRNA sequences and sequences of tRNA genes [13], which includes the Genomic tRNA Compilation, a compilation of cytoplasmic tRNA gene sequences derived from sequences of complete genomes included on DNA databases (approximately 7600 tRNA gene sequences from 131 complete genomes, covering bacteria, archaea, and higher and lower eukaryotes, published up to September 2004) and the Compilation of tRNA Genes, a summary of the published sequences of tRNA genes which were sequenced individually, including approximately 350 sequences of cytoplasmic tRNA genes that are not included in the Genomic tRNA Compilation.

The analysis of glycine tRNA gene sequences from mitochondria and chloroplasts used the Compilation of tRNA Genes section of the Compilation of tRNA sequences and sequences of tRNA genes [13] in conjunction with Mamit-tRNA: Compilation of mammalian mitochondrial tRNA genes [14], which currently contains 3064 tRNA gene sequences from 150 fully sequenced genomes available on GenBank database (NCBI).

All sequences were inspected manually in order to remove duplicates, with the result that each tRNA gene sequence is included in the respective analyses only once, unless the same sequence occurs in more than one species.

Figure 2A was adapted from an image generated by WebLogo software available from [40]. Figure 2B was taken from [14].

### Phylogenetic analysis

For the phylogenetic analysis, the same 466 glycine tRNA gene sequences (taken from [13]) were used as for the anticodon arm analysis. Clearcut software [41] was used to generate the relaxed neighbour joining tree files, which were visualized using FigTree software [42].

## Competing interests

The authors declare that they have no competing interests.

## Authors' contributions

HB formulated the hypothesis and carried out the sequence and phylogenetic analyses. WT played a mentoring role and provided original ideas. Both authors discussed ideas and wrote the manuscript.

## Reviewers' reports

### Reviewer 1: David Ardell, University of California, Merced

In this concise and well-written manuscript, Bernhardt and Tate present their case that the tRNA anticodon loop originated in part by duplication of a hypothetical NCCA 3' overhang of a primordial hairpin that was the ancestor of modern tRNAs. They assert that the universally conserved C35, C36 and A37 features of modern glycine tRNAs are therefore homologous to the CCA tail of modern tRNAs, and that – by implication (by the Principle of Continuity, using Crick's name for it) – glycine was the first amino acid encoded in the genetic code and assigned to GGN codons.

There are many reasons why this is a compelling argument, because, as documented by the authors, it synthesizes and rationalizes disparate evidence, observations and claims: that modern tRNAs originated by duplication of a primordial hairpin, that the position of tRNA introns after position 37 is well-conserved in all domains of life, that glycine is widely acknowledged to be encoded early in the genetic code, that experimentally, acceptor stem hairpins may be glycylated by modern-day glycyl-tRNA synthetases, and of course that glycine is universally encoded by GGN codons.

Additionally, although the authors do not raise this point, their hypothesis could explain the nucleotide distribution of the so-called "cardinal" nucleotide 37, 3' to the anticodon, which is nearly universally some modification of A, or much less frequently a modification of G, which is also a purine.

As compelling as all of this, we (the readers) must openly address the logic of the authors' arguments in the face of alternative possible explanations, and in particular, critically assess the direct evidence that they present for their case. We must also bring out tacit assumptions where necessary and attempt to evaluate them critically.

We are accustomed to applying well-understood models of sequence evolution to statistically evaluate similarity due to homology. However, in doing this with tRNAs we must be very careful, as these molecules violate the assumptions of these conventional methods – owing to their small size and intense functional constraint. For further discussion and reference of this issue for tRNAs please refer to Widmann et al [3].

The chief alternative explanation – to homology – for common features of different molecules is that they have common functional constraint. Of course, some – perhaps many – functionally constrained characters in tRNAs may ultimately be explained by common ancestry anyway. That is because they could have arisen by the "freezing" of specific random "accidents." By "accident" we simply mean that other variants of certain traits may have had at one time comparable fitness, but that then, through the evolutionary refinement and augmentation of function, other components of the biological system those traits interact with become co-adapted to specific variants, locking them in so that they could function well together. Thus, in a kind of biological symmetry breaking, historical accidents may become frozen and acquire novel functional constraint by exaptation.

**Authors' response**: *We would agree that random accidents provide 'opportunity' in the sense of "a combination of circumstances favourable for the purpose" *[43], *that then, through 'evolutionary refinement and augmentation of function', can indeed become functional constraints. In terms of the nascent anticodon-codon interaction and the subsequent evolution of coded protein synthesis, the proto-anticodon loop (N)CCA sequence created by the ligation of the two hairpins may have been absolutely required (the requirement for a purine in position 37 is discussed further in a later response to this reviewer). Coded protein synthesis was not predestined. As argued by others, it must have arisen in small, incremental steps, each of which had a selective advantage in itself (or, at least, was evolutionarily neutral) in order to be maintained. In fact, in terms of the functional requirements for the development of coded protein synthesis, a better descriptor than 'frozen accident' for what we are proposing might be 'necessary' or 'essential accident'. We acknowledge that coded protein synthesis could possibly have arisen by an alternative mechanism, in which case the presence of the (N)CCA sequence in the proto-anticodon loop may not have been required. However, we would assert that the appearance of this sequence constituted an essential pre-step for the advent of coded protein synthesis as it is seen today*.

The major evidence presented by the authors for their hypothesis is the nearly universal conservation of C35, C36 and A37 among glycyl tRNAs and identity of the CCA sequence with the CCA tail. They do not attempt any further demonstration of extended similarity of the anticodon loop to the 3' half of the acceptor stem, or indeed statistically reinforce the underlying specific hypothesis of tRNA origin by hairpin duplication due originally to Di Giulio. So their argument relies strongly on prior work, forcing us to evaluate some of that as well.

**Authors' response**: *Apart from the highly conserved CCA sequence, we have not found extended similarity of the anticodon loop to the 3' half of the acceptor stem in glycine tRNAs. However, it is perhaps interesting to note that a cloverleaf formed by the ligation of hairpins (as well as the hairpins themselves) based on the anticodon arm consensus sequence in Figure *2A, *i.e. with the sequence 5'-AGCU...GCC/UUNCCA-3', would be able to form the two terminal base pairs of the (acceptor) stem: U33-A38 and C/U32-G39 (underlined)*.

I must add at this point that the mitochondrial evidence that they present is entirely superfluous to their claims. The origin and divergence of mitochondria happened long after the origins of tRNA and the genetic code. Therefore, mitochondrial variation or lack thereof can shed little light on their hypothesis. Putting it another way, if mitochondrial glycyl tRNA genes lacked these features, this could have been written off as a derived peculiarity of mitochondria that neither supports nor undermines their claims.

**Authors' response**: *In response to this criticism, we have separated the analysis for cytoplasmic and organellar tRNAs. However, although mitochondrial sequences may not tell us about the origins of tRNA, the fact that the anticodon loop CCA **is **retained in these and chloroplast sequences enforces the argument that this sequence has indeed been 'frozen' in place in contemporary tRNA sequences: CC due to its fundamental importance in coding for glycine, and A37 due to its probable role in strengthening the anticodon-codon interaction through base stacking *[17].

Now surely, the absolute conservation of C35 and C36 nucleotides among glycyl-tRNAs is not surprising since there are no alternative genetic codes involving glycine.

**Authors' response**: *There are a number of alternative genetic codes, for example in Candida albicans *[38]* and the ciliates Tetrahymena thermophila and Paramecium tetraurelia *[39], *not to mention in mitochondrial genomes *[37]. *Interestingly, however, in none of these are the GGN glycine codons reassigned to another amino acid, indicating that the assignment of (N)CC anticodons to glycine has been frozen in place. In fact, this is true for the entire bottom row of the genetic code table, which contains codons with the sequence GNN, including the codons for alanine, aspartic acid, glutamic acid and valine as well as glycine, all of which are believed to be 'early' amino acids (see Table *1, *left column)*.

Furthermore, the near universality of A37 among glycyl-tRNAs is in fact superseded by the overwhelmingly high incidence of A37 in a majority of tRNA classes in eukarya and bacteria [15,44]. This so-called cardinal nucleotide has been discussed by Yarus [45] as playing a role in an "extended anticodon". Yarus showed that post-transcriptional modifications of A37 (and the minor variant G37) are correlated in *E. coli *with anticodon sequence, particularly position 36. This observation was generalized through sequence analysis to many more species of bacteria by Saks and Conery [44]. Archaea also always have a purine at this position, although the average frequency of G37 is much higher [15].

These modified purines at position 37 have a wealth of well-characterized functions in translation. They are involved in stabilizing the openness of the anticodon loop [46], constraining the motional dynamics of the anticodon stem-loop to better function in codon pairing [47], and stabilizing the interaction of position 36 with the first base of the codon [48]. Finally modified purines at position 37 are important for maintaining translational reading frame [49].

Do these facts invalidate the author's claims? Not necessarily, for the reasons I outlined above: historically once-arbitrary states of tRNAs may become functionally constrained through co-adaptation of other components of the translational apparatus. But until somebody demonstrates that translation could equally well have evolved with other nucleotides, say pyrimidines at the cardinal position instead of purines (more commonly A), the possibility remains that A37 in glycine is nearly inevitable for other reasons besides the one that the authors claim. Putting it another way, an important question is whether purines at position 37 are exaptations of a frozen accident or whether only purines could do the jobs they do in tRNAs.

**Authors' response**: *The fact that only purines could do the job they do at position 37 in no way invalidates our argument. No doubt A37 has been 'frozen' in place in contemporary tRNAs because it provides a compelling functional advantage in stabilizing the anticodon-codon interaction *[17]. *We believe it must have been in this position from the beginning, as it would have been in an anticodon loop formed in the way we have proposed by the ligation of two hairpins with 3'-CCA termini. Had it not, the anticodon-codon interaction, and indeed protein synthesis itself, may not have arisen. It would be hard to overstate the centrality of the anticodon-codon interaction, which is at the very heart of the mechanism of protein synthesis. A purine (A or G) in position 37 is required to stabilize this interaction and, in fact, enable it to occur. Equally, and as we have commented in the manuscript, an NCC anticodon was required for the establishment of the nascent anticodon-codon interaction due to hydrogen bonding considerations *[20]. *This can also be seen in the case of the glycine tRNAs from Staphylococcus aureus and Staphylococcus epidermidis which have been co-opted for the role of cell-wall synthesis and are unable to function in ribosomal protein synthesis *[16]: *they have a pyrimidine rather than a purine at position 37. Pertinent to this point, an analysis of the post-transcriptional modification of N37 is also instructive. It has been proposed that such modification serves to strengthen anticodon-codon interactions involving weaker A-U base pairs relative to those involving G-C base pairs *[50]. *Interestingly, of a set of post-transcriptionally modified tRNAs taken from three species where the structures of almost all tRNA sequences with their modifications have been elucidated (Escherichia coli (bacteria), Haloferax volcanii (archaea) and Saccharomyces cerevisiae (eukaryote cytoplasm)), none of the nine glycine tRNAs have a modified A37, the lowest proportion of any tRNA (Additional file *1, *part A). It has been suggested that early tRNA molecules contained no modified nucleotides *[8]. *If this is correct, the earliest anticodon-codon interactions would not have depended on post-transcriptional modification, with such modification possibly being introduced at a later stage of genetic code evolution (perhaps with the advent of protein enzymes) in order to utilize additional codons/amino acids. Significantly, the glycine tRNAs from the three organisms described above have the lowest average number (6.2) of post-transcriptionally modified nucleotides in the molecule as a whole compared with the other tRNAs (Additional file *1, *part B). While the fact that glycine tRNAs have a C at position 36 (the five tRNAs with the lowest proportion of modified N37s all belong to the bottom row of the genetic code table, meaning they possess C36) might provide an explanation for an unmodified nucleotide at position 37 *[45], *the relative lack of modification of the glycine tRNA molecule as a whole can not be explained on this basis. Despite relying on a slightly different argument, it suggests that glycine tRNA represents an early tRNA that did not require extensive post-transcriptional modification to function*.

Then what are we left with? I am a bit hungry for further demonstration of the underlying hypothesis that tRNAs originated by duplication of a primordial hairpin, ultimately ancestral to both the D and T arms of modern tRNAs. With this underlying hypothesis strengthened, one is more likely to consider seriously the authors' claims. The primordial hairpin duplication origin of tRNAs is a large and fairly complicated body of literature and I did not evaluate it exhaustively. I will focus on two papers that are easiest to understand because they most directly use well-understood methods: those are Di Giulio [51] and Widmann et al [3]. Di Giulio [51] used parsimony-based methods to study the similarity of the 3' and 5' halves of reconstructed ancestral tRNA sequences. Besides the intrinsic limitations of the parsimony analysis as noted in Widmann et al [3], there are biases in ancestral sequence reconstruction (ASR) with either parsimony or likelihood-based methods. In fact another study that used ASR to reconstruct ancestral tRNA sequences found that they did not even fold properly into the canonical secondary structure [52].

It is worth noting that objections of a quite similar nature have also been raised about the means by which Jordan et al [11] reported universal trends in amino acid compositional gain and loss in proteomes, which is cited by the authors in support of their hypothesis. These objections were raised by Goldstein and Pollock [53]. Incidentally, and perhaps in support of the authors, similar claims to Jordan et al [11] were made using entirely different methods and comparisons by Ivanov [54].

Widmann et al. [3] took an alternative approach to assess the question of paralogy of the two halves of the tRNA cloverleaf. They compared the distributions of similarity of actual extant 5' and 3' halves of tRNAs to those expected from a null model of random tRNAs. My chief objection to this otherwise excellent approach is that their null model of random tRNAs (generated by a shuffling procedure) are not filtered or verified to actually fold into a minimum free energy cloverleaf structure. It is possible that the requirements of this structure place additional constraints on sequence variation that would reduce the significance of their results.

**Authors' response**: *We would agree with the reviewer that the hairpin duplication origin of tRNA has not been proven, but we believe it is a credible hypothesis. Problems with phylogenetic analysis of tRNA may be due partly to a loss of evolutionary signal across 4 billion years of evolution in what are very short sequences of RNA *[3]. *We would also wish to make the following points*:

*1. The hairpin duplication theory for the origin of tRNA seems to us to be based primarily on two separate strands of evidence: the presence of a canonical intron insertion position between nucleotides 37 and 38 in the middle of the molecule, at the position one would expect if indeed tRNA were formed from two similarly-sized halves; and the experiments of Schimmel et al. demonstrating that hairpins containing 3'-CCA termini are able to be specifically aminoacylated by contemporary aminoacyl-tRNA synthetases, indicating, in light of the structural relationship between hairpins and the tRNA cloverleaf (see Figure *1*) and on the basis of the Principle of Continuity, that such hairpins were the precursors to tRNA. That these two lines of evidence so beautifully complement and support each other, we believe, adds considerable weight to the theory that tRNA originated from the duplication of hairpins that were able to be specifically aminoacylated at their 3'-CCA termini, and perhaps participated in some form of noncoded protein synthesis*.

*2. Point 1 notwithstanding, there is some disagreement in the literature as to whether tRNA was formed from a hairpin duplication or from the ligation of two different hairpins *[3]. *It should be noted that the ligation of two identical hairpins is not actually required for our argument. All that is necessary is that both hairpins contained 3'-CCA termini, one of which was incorporated into the nascent anticodon loop at the position we have proposed. The reason we have discussed the mechanism in terms of hairpin duplication is that it seems the simplest due to considerations of symmetry (see Figure *1). *As we know, however, nature is not always simple or symmetrical!*

In summary, the ideas in this paper are internally consistent and sound, and pull together many disparate facts. In my opinion, however, the exclusive evidence for the authors' specific claims is not overwhelmingly strong, and more could be done to bolster their claims.

### Reviewer 2: Rob Knight, University of Colorado

In this paper, Bernhardt and Tate propose an interesting new mechanism for the origin of the genetic code from primordial glycine tRNAs. Although speculative, the paper suggests that tRNAs might have had a monophyletic origin from duplication and divergence of tRNA-Gly, primarily because CCA occurs immediately upstream of the intron in the vast majority of tRNA-Gly sequences examined (this pattern could be due to the inclusion of the terminal CCA in a duplicated hairpin, as has been proposed by several authors). As Gly is encoded by GGN codons, this pattern would automatically introduce a glycine anticodon adjacent to the intron position. The authors conclude that glycine was the primordial tRNA, produced by hairpin duplication, and that other specificities arose from this original activity.

Several lines of evidence would make this argument more compelling.

First, although CCA is found next to the intron positions in a majority of tRNA-Gly sequences, this does not necessarily mean that this is the ancestral state. Building a phylogenetic tree of the tRNA sequences and demonstrating that the earliest-diverging branches have CCA would be reassuring (i.e. it is necessary to show that the earliest-diverging groups don't retain some rare but ancestral alternative). Rooting the tree is an issue if it is assumed that other tRNAs branch from within modern tRNA-Gly sequences, but several methods are available and should be used.

**Authors' response**: *In response to this helpful critique we have revised our manuscript to include Figure *3, *a phylogenetic tree of genomic glycine tRNAs. This figure shows that the 18 sequences not containing the anticodon loop CCA sequence are on isolated branches of the tree rather than spread throughout, indicating that this is a derived character, although the tree does not provide evidence of the ancestral glycine tRNA sequence*.

Second, some evidence that tRNA-Gly is the ancestral tRNA would be helpful. Again, trees could be built with a sample of tRNAs of different specificities. If other tRNA specificities branch from within tRNA-Gly, we would expect standard tests for monophyly on tRNA-Gly sequences to fail. If, however, tRNA-Gly sequences are monophyletic and branch from within some other specificity, the hypothesis would not be supported. Again, this analysis will be complicated by difficulties in rooting and in building a tree with so few characters, so a negative result will not be conclusive, but clear patterns, if obtained, could greatly aid in confirming or disconfirming the hypothesis.

**Authors' response**: *This has been attempted but without clear patterns emerging, and so the results have not been included in the paper. However, some support for glycine tRNA being the ancestral tRNA has been provided recently by Fujishima et al. (2008) *[55]. *Carrying out a phylogenetic/network analysis of 1953 archaeal tRNAs, they found that archaeal glycine tRNA might represent the ancestral sequence. Although starting from a different set of suppositions than ours (they believe that the split tRNA genes of Nanoarchaeum equitans represent the ancestral state of tRNA, rather than being derived from intron-containing tRNAs as we would argue), they conclude that 'minigenes encoding 5' and 3' tRNA sequence of tRNA^Gly ^were the origins of other tRNA genes in the very early stage of tRNA evolution'*.

Third, some justification for why CC rather than CA became the first fixed anticodon seems necessary (as both CC and CA would be present in all sequences duplicating the terminal CCA). There are several arguments based on thermodynamics and/or inspection of the canonical genetic code table that could be advanced here, although none stands out.

**Authors' response**: *Although we have not included with our proposal a possible sequence for the first anticodon loop, it seems reasonable to believe that the CCA sequence has always been in its current location, with (N)CC in the anticodon position. This is because seven nucleotide loops such as the anticodon loop interact predominantly through the central three nucleotides *[17]; *positions 35 and 36 seem to be particularly important for the strength of the anticodon-codon interaction, which with CCA in its current position are occupied by 'C's, providing two strong G-C interactions. Also, (as elaborated in our responses to the first reviewer), it appears likely that an A (or G) is required in position 37 in order to stabilize the anticodon-codon interaction by base stacking on the anticodon-codon helix *[17]. *Therefore CCA in its current position fulfills two important requirements for enabling a strong intermolecular interaction. In contrast, (C)CA in the anticodon position would probably have required a modified nucleotide in position 37 in order to stabilize the anticodon-codon interaction (due to the presence of an 'A' at position 36), as previously discussed. In the contemporary genetic code the (C)CA anticodon occurs in tryptophan tRNA, which usually has a modified nucleotide at position 37 (Additional file *1, *part A)*.

#### Some minor points

It might be useful to exclude chloroplasts and mitochondria on the grounds that we know these sequences are derived (from within the cyanobacteria and the alpha-proteobacteria respectively), and therefore cannot tell us about origins. However, examination of these sequences separately, because they can be rooted with outgroups from free-living bacteria, could be useful for testing whether the CCA is maintained by selection in modern tRNA-Gly sequences.

**Authors' response**: *As already noted, the mitochondrial and chloroplast data support the argument that the anticodon loop CCA sequence has been frozen in the vast majority of all glycine tRNAs. The fact that only 1 of 219 of the glycine tRNA gene sequences analysed from mitochondria and chloroplasts does not possess an anticodon CCA demonstrates this sequence has been maintained in these lineages*.

The issue with SELEX against glycine isn't coupling to the column (indeed, Gly columns are typically used as counterselections in amino acid selections), but rather the belief that a single H side-chain provides a target that will be very difficult to bind specifically given the steric issues with the linker and any protecting group used for the carboxyl or amine (depending on how the amino acid is coupled to the column).

**Authors' response**: *This point was originally unclear in the manuscript and has now been modified*.

Selections against a Gly column to the exclusion of other aminoacylated columns are thus not likely to generate aptamers that bind free Gly in solution with reasonable specificity. It is possible that this could be worked around using the Breaker lab's allosteric selection paradigm, which allows selection of sequences that bind targets free in solution, although this technique can only isolate very high-affinity binders that might not be relevant to the code's origin (much worse Kd's are available through the affinity chromatography approach). However, to my knowledge, these experiments have not been attempted.

### Reviewer 3: Eugene Koonin, National Center for Biotechnology Information, NIH

This article proposes a very specific hypothesis on the origin of the first steps in the evolution of the genetic code. According to Bernhardt and Tate, tRNA^Gly ^was the first tRNA to evolve, and more specifically, it evolved via the duplication of an RNA hairpin containing a 3'-CCA sequence and subsequent ligation of the two half-tRNAs that created the anticodon. The ligation, according to the hypothesis, was catalyzed by the evolutionary predecessor of the group I self-splicing intron that is present in the anticodon loop of tRNA^Gly^. The tRNA^Gly ^is supposed to have given rise to the rest of the tRNAs, presumably, via a series of duplications – these subsequent steps are not really discussed in the paper but rather implied by Figure 1.

**Authors' response**: *We have revised the manuscript to include mention of the evolution of the original glycine tRNA into tRNAs specific for other amino acids by a process of duplication and mutation*.

This may sound harsh but, for the sake of clarity, I will state my position in straightforward terms: to me, this is more of a free-wheeling speculation than a useful hypothesis. There are, I believe, three lines of argument marshalled in support of the hypothesis: i) glycine is widely believed to be one of the first, primordial amino acids, ii) according to Di Giulio's hypothesis, tRNAs evolved by duplication of "clover leaf halves", iii) tRNA^Gly ^contains a nearly universally conserved CCA sequence that is located in the anticodon loop and next to the universal intron insertion site. The first argument is reasonable but non-specific and weak; in any case, glycine could not have been the only primordial amino acid, and it is not at all clear why tRNA^Gly ^should have come first. The second argument that is presented in the present manuscript almost as an established fact is only a hypothesis itself, and not a particularly strong one; again, regardless of its validity, it says nothing about glycine specifically. The third argument is central to the article and is the only one that stems from actual sequence analysis. Unfortunately, the conservation of this CCA sequence is a simple consequence of the fact that all tRNA^Gly ^contain CC in the 2nd and 3rd positions of the anticodon, and this in turn is a straightforward consequence of the structure of the genetic code. Why glycine is encoded by GGX is an interesting question, and the answer may or may not be frozen accident but there seems to be no connection with the possible presence of the CCA-OH acceptor sequence in the primordial hairpin that gave rise to tRNA^Gly^. To me, this key proposal of the present paper is arbitrary.

The problems that, in my view, invalidate this paper are by no means unique, but rather, common to many ideas on the origin of life. The problem is extremely hard, and the temptation to engage in free speculation is strong and understandable. Unfortunately, this does not provide for a useful, let alone falsifiable hypothesis.

**Authors' response**: *Our responses to the first reviewer are relevant to the issues raised here. To summarize*:

*1. We are proposing that the first (glycine) tRNA arose within an environment containing up to 11 different RNA hairpins, each aminoacylated with a specific amino acid *[4]* (Table *1, *middle column). If tRNA has evolved from a single ancestral molecule, there must have been one that came first. Why glycine tRNA? As previously discussed, a number of theories on the origin of the genetic code (reviewed and summarized by Trifonov *[9]*), based on a range of suppositions, place glycine as the first, or in the first group of amino acids incorporated into the genetic code (see also *[10,11,19,20]).

*2. Schimmel's experimental work *[4]* on aminoacylatable hairpins supports and strengthens Di Giulio's theory of the hairpin origin of tRNA *[1,2,32]. *A large number of authors in addition to Di Giulio have proposed theories of the hairpin origin of tRNA. Of these, Ohnishi *[56], *and Nagaswamy and Fox *[57], *as well as Di Giulio *[32], *have proposed a hairpin ligation model with incorporation of the 3'-CCA terminus of the upstream hairpin in the anticodon loop of the resultant tRNA, however at different locations to our proposal*.

*3. Our finding of a highly conserved CCA sequence in the anticodon loop of 96% of contemporary glycine tRNA genes from bacteria, archaea and eukaryote cytoplasm forms the crux of our hypothesis. It seems to us that the presence and precise position of this sequence provides a possible clue to the identity of the first tRNA and the origin of the genetic code. Rather than being arbitrary, we would argue that our theory brings together a number of disparate theories/findings and produces a logical evolutionary scenario. Rather than being unfalsifiable, subsequent observations or experiments that gave proof that tRNA did not arise by a hairpin ligation, or that the canonical intron insertion position is not ancestral, or that glycine was not an early amino acid would all throw serious doubt on our proposal*.

*4. We agree with the reviewer that, 'Why glycine is encoded by GGX is an interesting question', and believe that our hypothesis offers a plausible explanation*.

## Supplementary Material

Additional file 1 Post-transcriptional modification of tRNAs from *E. coli, H. volcanii *and *S. cerevisiae*. Three (almost) complete sets of tRNA sequences with characterised modifications from *Escherichia coli *(bacteria), *Haloferax volcanii *(archaea) and *Saccharomyces cerevisiae *(eukaryote cytoplasm) were selected from the Compilation of tRNA sequences and sequences of tRNA genes from [13]. The order of their proposed utilization within the genetic code with their specific amino acids [9] is plotted against the number of post-transcriptionally modified nucleotides. **(A) **The percentage of tRNA sequences with a modified nucleotide in position 37. **(B) **The average total number of modified nucleotides in the tRNA molecule. The sequence sets included in the analysis contained between one and five tRNAs from an individual species and between three and twelve tRNAs from all three species. Note: the values for glutamine were not included due to the absence from the database of a sequence(s) from *S. cerevisiae*. Click here for file
